# New insights into the Lck-NF-κB signaling pathway

**DOI:** 10.3389/fcell.2023.1120747

**Published:** 2023-02-24

**Authors:** Jing Zhang, Yu-Jing Wu, Xiao-Xi Hu, Wei Wei

**Affiliations:** ^1^ Department of Pharmacy, The First Affiliated Hospital of Anhui Medical University, Hefei, China; ^2^ Key Laboratory of Anti-Inflammatory and Immune Medicine, Anhui Collaborative Innovation Center of Anti-Inflammatory and Immune Medicine, Ministry of Education, Institute of Clinical Pharmacology, Anhui Medical University, Hefei, China

**Keywords:** Lck, NF-κB, signaling pathway, autoimmune diseases, cancer

## Abstract

Lck is essential for the development, activity, and proliferation of T cells, which may contribute to pathological progression and development of human diseases, such as autoimmune disorders and cancers when functioning aberrantly. Nuclear factor-κB (NF-κB) was initially discovered as a factor bound to the κ light-chain immunoglobulin enhancer in the nuclei of activated B lymphocytes. Activation of the nuclear factor-κB pathway controls expression of several genes that are related to cell survival, apoptosis, and inflammation. Abnormal expression of Lck and nuclear factor-κB has been found in autoimmune diseases and malignancies, including rheumatoid arthritis, systemic lupus erythematosus, acute T cell lymphocytic leukemia, and human chronic lymphocytic leukemia, etc. Nuclear factor-κB inhibition is effective against autoimmune diseases and malignancies through blocking inflammatory responses, although it may lead to serious adverse reactions that are unexpected and unwanted. Further investigation of the biochemical and functional interactions between nuclear factor-κB and other signaling pathways may be helpful to prevent side-effects. This review aims to clarify the Lck-nuclear factor-κB signaling pathway, and provide a basis for identification of new targets and therapeutic approaches against autoimmune diseases and malignancies.

## 1 Introduction

Tyrosine kinases are involved in multiple cellular processes, including inflammation (Chichirau et al., 2019). Src, Tec and spleen tyrosine kinase (Syk) are three major kinase sub-classes that are closely related to T cell antigen receptor (TCR) signaling, which is the most important step for cells to recognize tissue damage and attack pathogens (Bradshaw, 2010). Over-activation of tyrosine kinases can change expression of genes, especially those encoding for cytokines that regulate the duration and degree of inflammation. The lymphocyte-specific protein tyrosine kinase (Lck), which is member of the Src family kinases (SFKs), is a non-receptor protein tyrosine kinase (PTK) with a molecular size of 56 kDa. Src family kinases are linked to nearly all stages of T-lymphocyte function and development; Lck is expressed in all T lineage cells and is vital for normal development of CD4 and CD8 single positive thymocytes, functional activity of memory and effector T-lymphocytes, as well as proliferation of naive mature T cells (Zamoyska et al., 2003). Lck is localized in the plasma membrane, through interactions with modified amino acid residues close to the N-terminus (Salmond et al., 2009). The Lck protein contains the following domains: N-terminal sites for myristoylation and palmitoylation, which are critical for membrane association and share little homology (Salmond et al., 2011); a unique region contains a di-cysteine motif that is required for association with the CD4 and CD8 coreceptors (Kim et al., 2003); a Src-homology 3 (SH3) and SH2 domains, which mediate intra- and inter-molecular protein-protein interactions *via* recognition of polyproline or phosphotyrosine motifs, respectively; and a SH1 and a C-terminal negative regulatory domain (Palacios and Weiss, 2004).

Studies have found that activated Lck is involved in the activation of nuclear factor κB (NF-κB) (Alqarni et al., 2022). NF-κB was initially discovered as a factor bound to the κ light-chain immunoglobulin enhancer in the nuclei of activated B lymphocytes (Sen and Baltimore, 1986). It is comprised of five subunits: RelA (p65), RelB, c-Rel, p105, and p100 (Hayden and Ghosh, 2011), p105 and p100 are processed to the mature NF-κB subunits p50 and p52, respectively, through selective degradation of their C-terminal IκB-like domain. NF-κB is a pivotal transcription factor in immunity and inflammation, cell survival and apoptosis, and cancer cell metastasis and invasion. NF-κB, which is induced during B cell maturation (Hayden and Ghosh, 2008; Baltimore, 2011), also plays a primary role in B cell development and activation. Abnormal expression of Lck and NF-κB can be found in autoimmune diseases and malignancies, including rheumatoid arthritis (RA) (Romagnoli et al., 2001; Herrington et al., 2016), systemic lupus erythematosus (SLE) (Matache et al., 2001; Barrera-Vargas et al., 2014), acute T cell lymphocytic leukemia (T-ALL) (Han et al., 2014; Gao et al., 2019), and human chronic lymphocytic leukemia (CLL) (Shinohara et al., 2005; Zanesi et al., 2012). In addition, Lck expression is increased in numerous solid tumors, such as glioma (Summy and Gallick, 2003; Du et al., 2009; Kim et al., 2010; Zepecki et al., 2019), cholangiocarcinoma (Pei et al., 2015; Sugihara et al., 2018; Conboy et al., 2023), and breast cancer (Bai et al., 2019; Meng et al., 2021), etc. Although targeting NF-κB may be a useful strategy for therapeutic intervention against inflammatory diseases, it is also involved in normal cellular physiology, such as immune responses, embryonic development of the limbs, bones, skin, and other organs (Li and Verma, 2002). Global inhibition of NF-κB may thus lead to serious adverse reactions, hence it is urgent to explore the functional interactions between NF-κB and other signaling pathways. Anti-inflammatory-based therapies with monoclonal antibodies have been safe and efficient for the treatment of inflammatory disorders, albeit at great expense. Several novel molecular targets, such as kinases, have now been considered as key mediators in multiple pathological conditions (Singh et al., 2016). In this review, we focus on the Lck-NF-κB signaling pathway, with the aim to determine potential molecules or targets to improve treatment against autoimmune diseases and malignancies.

## 2 Function and normal activation of Lck and NF-κB

### 2.1 Function and activation of Lck

Regulation of Lck functionality and activity is strictly controlled by phosphorylation or dephosphorylation of tyrosine residue 394 and 505 (Tyr^394^, Tyr^505^) (Philipsen et al., 2017), or by conformational transitions because of ligands binding to the SH2 domains and/or SH3 domains of the kinase. Tyr^505^ inhibits kinase function and induces a closed/inactive form of the enzyme when phosphorylated by C-terminal Src kinase (Csk). Upon stimulation, recruitment of Lck and dephosphorylation of Tyr505 by CD45 results in an open conformation and exposure of the activation loop (A-loop), which contains the activating tyrosine residue. Trans-phosphorylation of Tyr394 within the A-loop of the kinase domain promotes an active conformation and enhances the catalytic activity by 2-4 fold. Therefore, the interactions of the SH2/SH3 domains and phosphorylation of the tyrosine in the activation loop control Lck conformation and activity (Palacios and Weiss, 2004). Lck is mostly localized in a non-uniform way in plasma membrane microdomains packed with cholesterol and glycosylphosphatidylinositol (GPI)-anchored proteins, where the activated TCR and associated signaling molecules assemble (Salmond et al., 2009). This specific location of Lck is coincident with its vital role as an early player in both the TCR/CD3 complex and CD4 signaling pathways. Lck can phosphorylate the immunoreceptor tyrosine-based activation (ITAM) motifs of the TCR/CD3 complex, resulting in changes in intracellular free calcium concentration [(Ca2^+)^i] and mediating gene expression alterations, which are critical for T cell survival and activation (Palacios and Weiss, 2004). In the course of T cell activation, Lck acts both as an adaptor molecule and a tyrosine kinase, which binds and phosphorylates a great number of cell-surface and intracellular substrates. Lck-deficient mice are characterized by an incomplete block in the differentiation of CD4^−^CD8^−^CD44^−^CD25^+^ progenitor cells, where pre-TCR signaling is required for transition to the next developmental stage, indicating the vital role of Lck in pre-TCR signaling. Impaired maturation of thymic CD4^+^ and CD8^+^ cells causes a lack of mature peripheral T cells in *Lck* ^*−/−*^ mice. Additional research also revealed a severe reduction in the size of the thymus in Lck-deficient mice, mainly owing to a 10-fold decrease in the number of double-positive thymocytes (Zamoyska et al., 2003). Taken together, these findings show the essential role of Lck in T cell development.

### 2.2 Function and activation of NF-κB

NF-κB has been found to promote the expression of chemokines, cytokines and receptors that recognize TCR, intercleukin 2 (IL-2), IL-6, IL-8, and tumor necrosis factor-α (TNF-α). NF-κB can bind to enhancers and promoters of its target genes, such as matrix metallopeptidase 9 (MMP-9), vascular endothelial growth factor (VEGF), urokinase type plasminogen activator (uPA) and chemokine receptors (CXCR1, CXCR2, CXCR3, CXCR4, CCR2 and CCR7, etc.) that control metastasis, invasion and angiogenesis of cancer cells (Richmond, 2002; Taniguchi and Karin, 2018; Tatangelo et al., 2022). In addition, survival and maturation of B lymphocytes by BAFF/BAFF receptor (BAFFR) are mainly mediated by NF-κB (Zhang et al., 2017). In T lymphocytes, a large number of cytokines and their receptors that regulate proliferation, survival or specialization into functional subsets control or are controlled by NF-κB (Gerondakis and Siebenlist, 2010). NF-κB is critical for the regulation of CD4^+^ T cell differentiation, especially of T helper cells (Th)17 and regulatory T cells (Treg). c-Rel and RelA can directly regulate differentiation of Th17 cells through inducing RORγt expression, which acts as a Th17 cell specific transcription factor (Oh and Ghosh, 2013). Early studies have shown that the frequency of Treg cells in mutant mice lacking IκB kinase *β* (IKKβ) or p50 and c-Rel was reduced, indicating a cell-intrinsic role for the canonical NF-κB pathway in the development of Treg cells (Sun et al., 2013).

NF-κB is normally sequestered in the cytoplasm through close association with the NF-κB inhibitor IκBα and p100 proteins. NF-κB may be activated by numerous stimuli, such as cytokines, viruses, and immune-related stimuli (Wan and Lenardo, 2010). In the process of NF-κB activation, the IKK holoenzyme phosphorylates IκB, resulting in its ubiquitination and proteasomal degradation. NF-κB heterodimers, which are composed by p50, p65, or c-Rel, are released from IκB, translocate from the cytoplasm to the nucleus and regulate expression of specific genes. In addition, IKKα can also be phosphorylated and activated directly by NF-κB-inducing kinase (NIK) (Hsieh et al., 2004), leading to the phosphorylation and ubiquitination of p100, partially processing p100 into p52, resulting in the translocation of p52/RelB heterodimer into nucleus. Activation of the NF-κB pathway controls the expression of many target genes that are related to cell survival, apoptosis, and inflammation.

### 2.3 Lck-NF-κB signaling pathway in physiology

**FIGURE 1:**
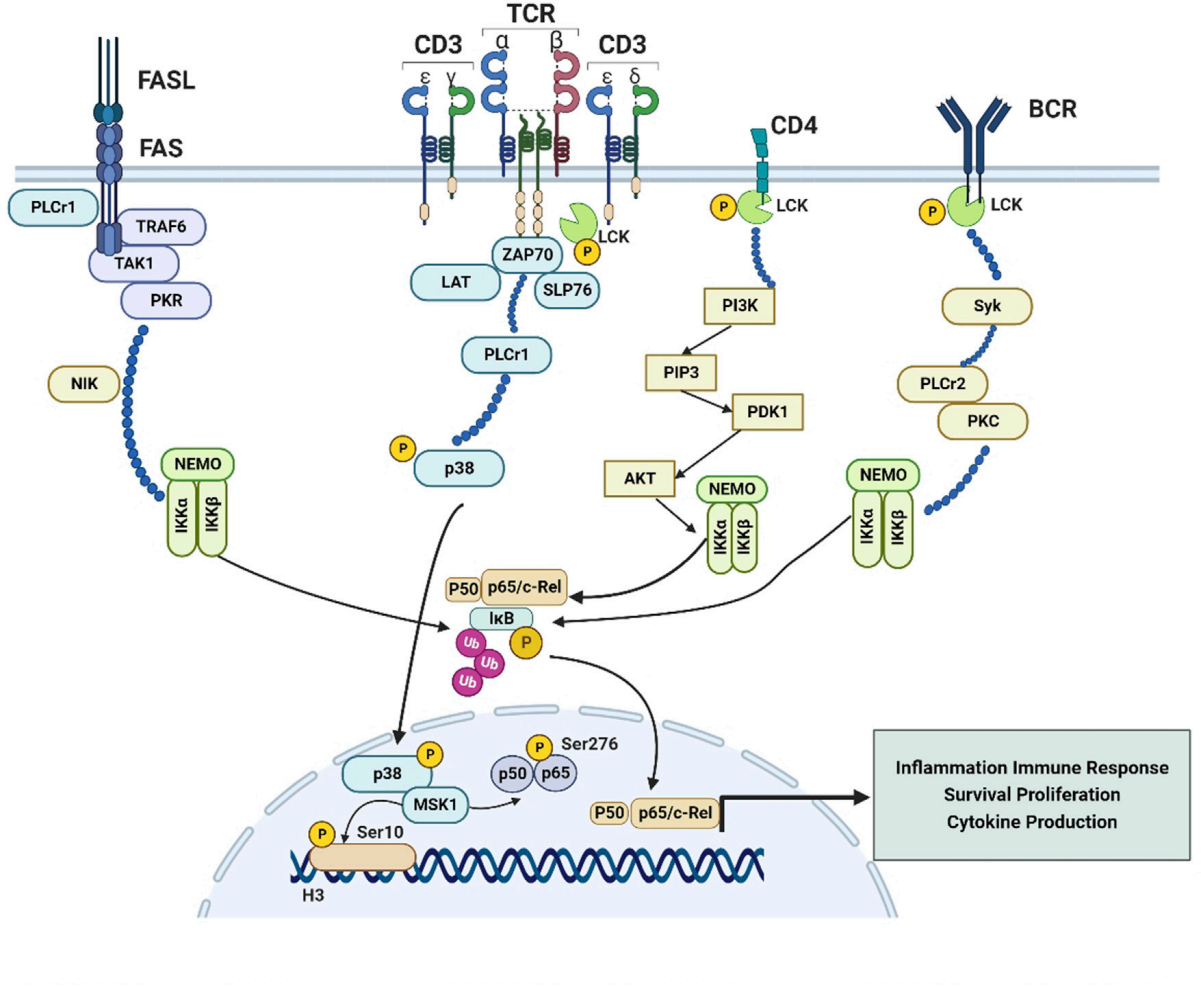
The Lck-NF-κB signaling pathway. Exploration of Lck-NF-κB signaling pathway is of great importance for finding a new target of autoimmune diseases and malignancies treatment. 1. In T cells, Lck is recruited to the TCR/CD3 complex, resulting in the recruitment, phosphorylation and activation of ZAP70, activated ZAP-70 in turn phosphorylates T-cell-specific adapters, such as the LAT and SLP-76, as well as the activation of PLCγ1, which can induce the activation of Ras and MAPK, then the MAPK p38 could govern an autoregulatory loop that affects a series of genes transcribed by NF-κB in response to inflammatory stimuli, and induce phosphorylation of histone H3 at serine 10 through MSK1 or other histone kinases, further increased the DNA accessibility for NF-κB binding at some specific promoters, like IL-12p40 and IL-6; 2. MSK1 activation also increases transcription through p65 phosphorylation at serine 276 Fas-induced death in T cells is strongly dependent on activation of PLCγ1, Striking upregulation TAK1 and TRAF6 results in activation of the NF-κB pathway. Transcriptional upregulation of TRAF6 and PKR was supported by increased expression of NIK in all Fas-positive cells, ultimately leading to activation of IKKα; 3. Lck is connected to the activation of PI3K, activated PI3K could transform PIP2 into PIP3, then PIP3 bound to Akt and PDK1 through pleckstrin homology (PH) domains, colocalization of Akt and PDK1 induced by PIP3-Akt binding induced PDK1 to phosphorylate Akt, then activated Akt in turn induced the activation of NF-κB through utilization of IKK complex; 4. In the B cells, Lck has a vital role in potentiating BCR signaling, then induces the activation of Syk, which is an essential mediator for PLCγ2 activation during BCR engagement, and eventually leads to the activation of IKK and NF-κB. Activation of the NF-κB pathway induced by BCR is associated with PKC and PLCγ2.

Lck is usually involved in the TCR signaling initiation, which determines T cell antigen specificity (Kowallik et al., 2020). The TCR is inactive in resting conditions, while it stabilized in an active conformation upon ligand binding; Lck is then recruited to the TCR/CD3 complex, binding to CD3ζ resulting in its activation through Y394 phosphorylation. Phosphorylation of ITAMs by Lck initiates TCR signaling. Phosphorylated ITAMs act as docking sites for the tandem SH2 domains of Zeta-chain-associated protein kinase 70 (Zap-70), resulting in its recruitment, phosphorylation and activation, thus orchestrating T cell differentiation and proliferation (Nika et al., 2010). Activated ZAP-70 in turn phosphorylates T-cell-specific adaptor proteins, such as the adaptor molecule linker for activation of T cells (LAT) and SH2 domain containing leukocyte protein of 76 kDa (SLP-76) (Simeoni, 2017), contributing to the recruitment and activation of further downstream enzymes and kinases, then leading to secondary messenger generation and culmination of T cell activation. LAT contains four phosphorylation sites for Zap70: Y132, Y171, Y191, and Y226; Phosphorylation of Y132 recruits phospholipase C-γ1 (PLCγ1) to promote calcium influx and activation of the Ras-Mitogen-Activated Protein Kinase (MAPK) pathway (Zhang et al., 2000; Balagopalan et al., 2010). MAPK p38 regulates an autoregulatory loop that affects a group of genes transcribed by NF-κB in response to inflammatory stimuli. MAPK p38 has the ability to induce phosphorylation of histone H3 at serine 10 through mitogen- and stress-activated kinase 1 (MSK1) or other histone kinases, and further increased DNA accessibility for NF-κB binding sites at specific promoters, such as IL-12p40 and IL-6. MSK1 activation also increases transcription through p65 phosphorylation at serine 276, indicating that p38-dependent H3 phosphorylation may mark some promoters for the increased NF-κB recruitment (Ghosh and Hayden, 2008) (Figure 1).

The Fas receptor, a member of TNF-α family of death receptors, is reported to mediate T-cell responses. Akimzhanov et al. (2010) reported that Lck could mediate the activation of the Fas signaling pathway, Lck-deficient cells were absolutely resistant to the apoptosis induced by Fas. And Fas signaling is associated with an increase in the concentration of cytoplasmic calcium, due to increased calcium oscillation in the cytoplasm by PLC-γ1 activation. It has been reported that, in addition to inducing apoptosis, Fas signaling can also activates pathways involved in stress response and cell survival, including ERKs, p38 and NF-κB (Oh et al., 2012). Striking upregulation of several factors, including TGF-β-activated kinase-1 (TAK1), and TNF receptor-associated factor-6 (TRAF6), which are resistance to Fas receptor-mediated apoptosis, can be found in the hematopoietic progenitors, ultimately resulting in activation of NF-κB pathway (Mizrahi et al., 2014). The canonical pathway involving TAK1 activation was upregulated in Fas-positive cells, which was responsible for direct activation of NF-κB. The alternative pathway is characterized by transcriptional upregulation of TRAF6 and double-stranded RNA-activated protein kinase (PKR), which was supported by increased expression of NF-κB-inducing kinase (NIK) in all Fas-positive cells (Zamanian-Daryoush et al., 2000). Both pathways lead to activation of the α-catalytic subunit of IKKα, which is a determinant of NF-κB activity through phosphorylation and detachment of IκB and processing of the NF-κB precursors p100 and p105 to p52 and p50 (Hoffmann and Baltimore, 2006) (Figure 1).

It has been reported that SFKs were associated with the activation of phosphatidylinositol-3-kinase (PI3K), while the reduced PI3K activity found in Nijmegen breakage syndrome (NBS) lymphoblasts was linked to impaired expression of Lck (Sagan et al., 2008). Studies also pointed that TCR-induced tyrosine phosphorylation of Tyr688 in the p85 subunit of PI3K and the following activation of PI3K were connected to the presence of Lck. Activated PI3K could transform phosphatidylinositol 4,5-biphosphate (PIP2) into phosphatidylinositol 3,4,5-triphosphate (PIP3), a secondary messenger that resulted in the direct activation of Akt. Subsequently, PIP3 bound to Akt and the Akt activator 3-phosphoinositide-dependent kinase 1 (PDK1) through pleckstrin homology (PH) domains, leading to phosphorylation of Akt at Tyr308 in the activation loop (Sun et al., 2010). Activated Akt in turn induced the activation of NF-κB through utilization of IKK and activation of MAPK p38 (Figure 1).

Lck in B cell receptor (BCR)-stimulated chronic CLL cells could catalyze the proximal phosphorylation of CD79a, which forms a dimer associated with membrane-bound immunoglobulin together with the related CD79b protein, thus forming the BCR complex. Lck activation in B cells induces distal signals including phosphorylation of Syk, activation of PI3K/Akt, MAPK and NF-κB signaling pathways and enhanced cell survival. The levels of induced pERK, pAkt and pIKK were higher in BCR-stimulated CLL cells expressing high levels of Lck than those expressing low Lck levels (Talab et al., 2013). Lck is a vital participant during the induction of Syk activity in CLL cells, and Syk is an essential mediator for PLCγ2 activation during BCR engagement. Activation of the NF-κB pathway induced by BCR is known to be associated with protein kinase Cβ (PKC) and PLCγ2 (Shinohara et al., 2005), hence Lck may induce the activation of NF-κB through the Syk-PLCγ2 pathway (Figure 1).

## 3 Abnormal Lck-NF-κB signaling and treatment in diseases

### 3.1 Lck-NF-κB signaling in autoimmune diseases

#### 3.1.1 Lck-NF-κB signaling in RA

Lck is an essential factor for T cell maturation and activation, as well as for proliferation (Gascoigne and Palmer, 2011). It is responsible for signaling activation through TCR, which can further lead to upregulation of inflammatory cytokines, including IL-2 and interferon-γ (IFN-γ). It then contributes to T lymphocyte activation and proliferation, ultimately generating an immune response (Majolini et al., 1999). Our group found that the Lck specific inhibitor A770041, a pyrazolopyrimidine inhibitor, could significantly prevent abnormal proliferation and activation of CD4^+^ T (CD4^+^CD69^+^, CD4^+^CD154^+^) cells induced by IgD (Wu et al., 2018). Hence, inhibition of Lck is likely to generate an immunosuppression effect that could be exploited for the treatment of T-cell-mediated diseases, such as RA, SLE, inflammatory bowel disease, type 1 diabetes, organ graft rejection, and others (Kamens et al., 2001) (Table 1).

**TABLE 1:** The role of Lck in different diseases.

Diseases	Alteration	Biological effect	References
Lck in autoimmune diseases			
RA	Activation of Lck and NF-κB	Abnormal proliferation and activation of CD4^+^ T cell, inflammation infiltrating, activation of MMPs, DCs, macrophages	Couzin-Frankel (2013), Herrington et al. (2016), Briseno et al. (2017), Ronin et al. (2019)
SLE	Increased ubiquination and reduced expression of Lck, NF-κB activation	FcRε instead of CD3ε, Syk instead of ZAP70, Syk activation and stronger Syk binding to LAT, PLC-γ1, increased pro-inflammatory cytokines and decreased DCs maturation	Livolsi et al. (2001), Fan et al. (2003), Jury et al. (2003)
Lck in hematologic malignancies			
T- ALL	High level of Lck, NF-κB activation	Tumor growth, neoplastic transformation of HTLV-I infected T-cells, JAK/STAT signal activation	Kamens et al. (2001), Harr et al. (2010), Chen and Chen (2015)
CML	High level of Lck	T cell proliferation and activation, pro-inflammatory cytokine production	Kamens et al. (2001), Ulivieri et al. (2003)
CLL	High level of Lck (not all) and activation of NF-κB	Facilitate calcium signaling, monoclonal B cell proliferation, increased IL-6, Syk phosphorylation, apoptosis defects, activated MAPK and PI3K/Akt signaling pathway	Jin et al. (2003), Han et al. (2022)
Lck in solid cancers			
Glioma	Increased Lck expression and activation	Lck inhibition suppresses of gloima cell and CD133^+^ cell expansion, reduces self-renewal-related proteins expression, controls glioma cell migration	Pei et al. (2015), Conboy et al. (2023)
Cholangiocarcinoma (CCA)	Increased Lck expression	Lck inhibition reduces YAP transcriptional activity and tyrosine phosphorylation, downregulates proliferation and upregulates apoptosis of CCA tumor cells	Sugihara et al. (2018), Bai et al. (2019)
Breast cancer	Lck overexpression	CD4^+^ T cells, CD8^+^ T cells, B cells, and dendritic cells infiltration, Lck inhibitor reduces bone metastases	Li and Verma (2002), Samraj et al. (2006), Singh et al. (2016)

As elucidation of the vital role of the immune system against malignancies has contributed to new discoveries in immunotherapy, research is now focused on NF-κB inhibition to fight autoimmune diseases, including RA, SLE, and multiple sclerosis (Couzin-Frankel, 2013). In the pathogenesis of RA, T and B cells, monocytes, and neutrophils migrate from the circulation to the joints under inflammatory conditions (Sehnert et al., 2020). Constitutive NF-κB activation and increased NF-κB DNA binding activity have been observed in the synovium, which is essential for proinflammatory gene expression associated with cartilage destruction, synovium hyperplasia, pannus formation, and lymphocyte recruitment (Herrington et al., 2016). In the collagen-induced arthritis (CIA) mouse model, both c-Rel-deficient and p50-deficient mice showed impaired humoral and cellular immunity to type II collagen and failed to develop CIA. Interestingly, in acute arthritis, c-Rel-deficient mice had normal response, while p50-deficient mice were refractory to acute arthritis induction, suggesting that c-Rel is not necessary for joint destruction, while p50 is essential for local joint inflammation and destruction (Campbell et al., 2000). NF-κB promotes autoimmune T cell activation through modulating the function of dendritic cells (DCs). Activated DCs, associated with proinflammatory cytokine production and costimulatory molecule expression, contribute to the pathogenesis of RA (Pai and Thomas, 2008). This NF-κB activation process in RA involves several molecules, including TCRs and members of the toll-like receptor (TLR) family. These molecules can activate macrophages in the synovium, resulting in IκB phosphorylation and formation of NF-κB dimers, such as p50/p65, which induce expression of numerous pro-inflammatory chemokines and cytokines including IL-1, IL-6, IL-8 and TNF-α. These cytokines cause massive infiltration of immune cells into the synovium. In response to TNF-α or IL-1, fibroblast-like synoviocytes (FLSs) express a great number of NF-κB-induced genes, including MMPs that contribute to joint destruction and chemokines, which may perpetuate inflammation (Simmonds and Foxwell, 2008).

It may be possible to reduce systemic toxicity through targeting specific NF-κB subunits or signaling components that contribute to particular diseases. RelA plays an important role in Treg biology, because specific deletion of RelA resulted in systemic, spontaneous, and severe autoimmune syndrome (Gerondakis and Siebenlist, 2010). RelA-deficient Treg cells produced inflammatory cytokines (IFN-γ, IL-4, IL-17, IL-6, and TNF-α), and showed reduced self-stability and expression of Foxp3. RelA-deficient mice developed serious autoimmune syndrome, along with highly lymphoid and myeloid cell activation and multi-organ immune infiltration (Ronin et al., 2019). RelB was one of the earliest transcription factors to be associated with development of DCs (Briseno et al., 2017). RelB knockout mice showed a decreased DCs population, and exhibited significant myeloid expansion of immature polymorphonuclear neutrophils (PMNs), resulting in T-cell dependent autoimmunity, splenomegaly, and impaired lymph node development (Weih et al., 1996). Furthermore, deficiency of RelB in DCs in C57BL/6 mice lead to systemic and spontaneous accumulation of Foxp3^+^ Treg cells, further impairing oral tolerance induction and resulting in an obvious Th2 bias among accumulated Foxp3^+^ Treg cells (Andreas et al., 2019). Novack et al. (2003) found that NIK deletion resulted in p100 accumulation in osteoclast precursors, which impaired osteoclast differentiation *in vitro* through sequestration of the RelB/p100 complex in the cytoplasm; Importantly, p100 (NFKB2) blockade was able to restore the impaired osteoclastogenesis in *NIK* ^−/−^ precursors (Vaira et al., 2008). In addition, TNF-α induced p100 accumulation in osteoclast precursors, and osteoclast formation induced by TNF-α was markedly increased in NFKB2 knockout mice (Tanaka and NakanoNF-kappaB2, 1002009). Therefore, p100 blockade may be a novel strategy for the treatment of bone diseases such as RA, since osteoclast formation induced by TNF-α is crucial for the progression of bone diseases. IKKβ can regulate NF-κB activity in many cell types, including FLS, and plays a crucial part in the production of matrix metalloproteinases, adhesion molecules and cytokines in FLS. Aupperle et al. (2001) reported that overexpression of wild-type (wt) IKKβ clearly increased IL-6 and IL-8 production in synoviocytes, while expression of a dominant negative (dn) form of IKKβ abolished IL-6 and IL-8 induction. In a rat adjuvant arthritis model, IKKβ activation in the synovium induced by wt IKKβ caused clinical arthritis in healthy rats, while IKK activity suppression through dn IKKβ diminished arthritis (Hu et al., 1999). IKKε also exerts an inflammatory response in RA through activating the NF-κB signaling pathway (Cavalcante et al., 2010). The IKKε inhibitor amlexanox significantly limited the inflammatory activation in RA (Zhou et al., 2018). IKKε knockout mice showed reduced expression of IL-1β, IL-6, TNF-α and IFN-γ through regulation of the NF-κB signaling pathway, thus mediating the RA inflammatory response.

#### 3.1.2 Lck-NF-κB signaling in SLE

**FIGURE 2:**
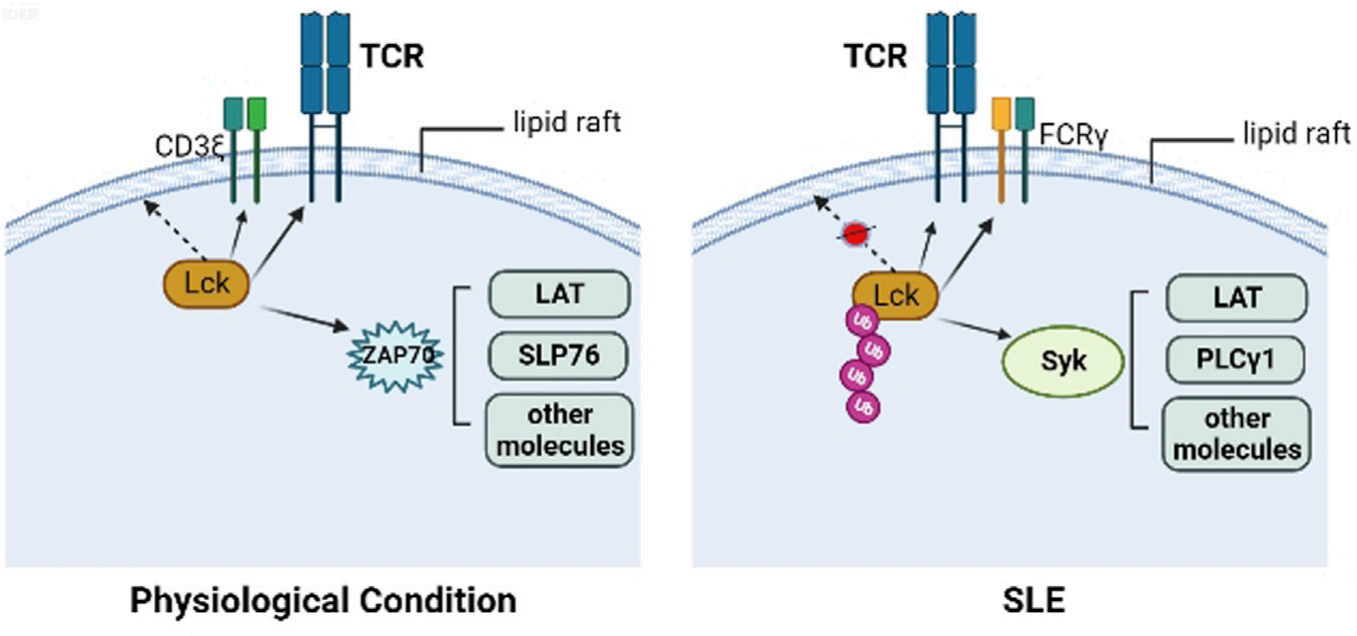
Lck in physiological condition and SLE. In the physiological condition, Lck is S-acylated in its N-terminal domain, then localized at the lipid rafts, ZAP70 is recruited to the phosphorylated ITAMs in CD3ζ chain, then is phosphorylated by Lck and activates other molecules, including LAT, SLP76. In the T cells from patients with SLE, the reduced Lck expression in lipid rafts was found because of its increased ubiquitination, along with FcRγ substituting CD3ζ, and recruiting Syk instead of ZAP-70. Activation of Syk does not require Lck phosphorylation, and Syk shows stronger binding to LAT, PLC-γ1, and other molecules in SLE patients compared with healthy controls.

SLE is a systemic autoimmune disease which is characterized by dysregulated cellular, innate, and humoral immune responses. Chronic inflammation in SLE involves multiple organs, such as the kidney, skin, joints and blood vessels. Kidney inflammation in particular can result in lupus nephritis, which is the main reason for morbidity and mortality (Moulton et al., 2017). Abnormal activity and expression of Lck has been found in lymphocytes from patients with SLE. There are many defects in TCR-associated PTK activity in SLE patients, including substitution of the CD3ζ chain with the common γ chain of the Fcε receptor (FcR), which is similar to CD3ζ, and signaling through Syk instead of ZAP-70 (Enyedy et al., 2001). Activation of Syk does not require Lck phosphorylation, and Syk shows stronger binding to LAT, PLC-γ1, and other molecules in SLE patients compared with healthy controls. The abnormal expression and activity in Lck and ZAP-70 may contributes to the loss of tolerance, and finally results in autoimmunity (Barrera-Vargas et al., 2014). Under physiological circumstances, Lck is S-acylated in its N-terminal domain, then localized at the lipid rafts (Hawash et al., 2002), while the reduced Lck expression in lipid rafts was found in the T cells from patients with SLE because of its increased ubiquitination (Jury et al., 2003). Previous studies found that atorvastatin could reverse the lipid raft-associated signaling abnormalities in lupus T cells, for example, it could increase the total amount of Lck in the lipid raft domains, reduce the association between Lck and CD45 which contributes to the Lck activation, and regulate proximal TCR signaling (Jury et al., 2006). Therefore, restoration of the association between Lck and lipid rafts may rescue the aberrant T cell signaling observed in patients with SLE (Figure 2).

It has been reported that deficiency in peripheral tolerance to self-antigens is critical for SLE pathogenesis, while DCs are essential for maintaining peripheral tolerance through blocking activation of self-reactive T cells. Kalergis et al. (2009) reported that most stimuli that drive DC maturation induced transcription of NF-κB-controlled genes, which suggests that NF-κB is a key transcription factor in determining the DC phenotype. Inhibition of NF-κB activation has been considered as an effective strategy for maintaining DCs in an immature state to promote immune tolerance, thus suppressing SLE pathogenesis. In addition, osteoporosis (OP), characterized by increased bone fragility, decreased bone mineralization and increased fracture risk, has been common in patients with SLE. Activation of NF-κB in SLE patients inhibited osteogenic differentiation induced by BMP-2 through preventing Smad protein binding to its target genes and blunt Smad signaling, which suggests that inhibition of NF-κB can be a novel approach to increase osteoblastic differentiation for OP treatment in SLE patients (Tang et al., 2013).

Autoimmune diseases are characterized by increased production of proinflammatory cytokines including IL-1, IL-6, and TNF-α, which has been related to the activity of NF-κB. NF-κB activation is linked to IκB phosphorylation and degradation, and SFK activity has been regulated by serine and tyrosine phosphorylation of IκB. First, some studies reported that the PTK inhibitor genistein and the specific Src kinase inhibitor damnacanthal significantly inhibited serine phosphorylation and subsequent degradation of IκB-α, and the translocation of p65 from the cytoplasm to the nucleus. Therefore, the Src tyrosine kinases cSrc and Lck contribute to the activation of serine kinases such as IKK1 or IKK2, which can lead to phosphorylation and degradation of IκB-α in response to proinflammatory stimuli, resulting in NF-κB activation. Second, Livolsi reported that inhibition of Src kinases, such as Lck and Zap-70, prevented both NF-κB activation and tyrosine phosphorylation of IκB-α (Livolsi et al., 2001), suggesting a close relationship between NF-κB activation and Src-mediated tyrosine phosphorylation of IκB-α. Fan et al. (2003) also reported that tyrosine phosphorylation of IκB-α and NF-κB activation were significantly reduced in cSrc knockout cell lines treated with hypoxia/reoxygenation, suggesting that cSrc or Lck are probably responsible for direct IκB-α phosphorylation. Thus, Src tyrosine kinases, such as Lck, may be useful targets for the treatment of autoimmune diseases through preventing NF-κB activation.

Abnormal activation of both Lck and NF-κB can be found in autoimmune diseases, such as RA and SLE, and several signaling pathways may result in NF-κB activation. Therefore, we interrogated whether abnormal activation of NF-κB induced by Lck may contribute to the pathogenesis of RA or SLE and whether Lck may be an appropriate target in the Lck-NF-κB signaling for the treatment of autoimmune diseases. A770041, a selective inhibitor of Lck, prevented activation and proliferation of T cells, as well as allograft rejection in transplanted hearts, suggesting that inhibition of Lck may be considered as an efficient approach in treating inflammatory diseases, including RA, SLE, multiple sclerosis, and type 1 diabetes, and preventing organ transplant rejection (Stachlewitz et al., 2005); The novel selective and potent inhibitor of Lck 2-aminopyrimidine carbamate has been proved to inhibit human T cell proliferation, thus possibly serving as a useful immunosuppressive for the treatment of T cell-mediated autoimmune diseases or graft rejection (Martin et al., 2006); BMS-243117, a new benzothiazole template, is an efficient and selective Lck inhibitor with excellent activity *in vitro* and good potency in T cell proliferation assays (Das et al., 2003). All these pieces of evidence suggest that it is certainly worth exploring the Lck-NF-κB signaling pathway for the treatment of autoimmune diseases, especially targeting Lck.

### 3.2 Lck-NF-κB signaling in cancer

#### 3.2.1 Lck-NF-κB signaling in hematological malignancies

A great number of studies have reported that abnormal expression and regulation of Lck was closely related to cell carcinogenesis. For example, overexpression of Lck in transgenic mice resulted in thymic tumors (Abraham et al., 1991), and Lck was found to be aberrantly activated in human lymphocytic and non-lymphocytic malignancies (Majolini et al., 1999). Numerous human and murine lines derived from T cell leukemias, including T-ALL, showed abnormally high levels of Lck expression, which was associated with hyperphosphorylation of cellular proteins. The oncogenically activated Lck mutant in T-ALL derived cell lines could lead to neoplastic transformation of human T cell leukemia virus type I (HTLV-I) infected T cells. Moreover, the IL-2-independent growth of T cells transformed with HTLV-I was associated with constitutive activation and phosphorylation of the JAK/STAT kinase, suggesting the possible relationship between overexpression of Lck and activation of the JAK/STAT signaling pathway. Constitutive activation of NF-κB was also observed in human T-ALL and mouse models with acute T cell leukemia (Dos et al., 2010). NF-κB plays a significant pro-oncogenic role in leukemic T cells and modulates T cell leukemogenesis through affecting microenvironmental stromal cells. Inhibition of the canonical NF-κB signaling abolished leukemic T cell growth (Staal and Langerak, 2008). Studies also reported that Lck interacted with inositol 1,4,5-Trisphosphate (IP3) receptors to facilitate calcium signaling. Calcium-dependent activation of calcineurin was the integral step to inhibit apoptosis induced by glucocorticoids, and Lck blocking enhanced glucocorticoid sensitivity and apoptosis in lymphoid cell lines and chronic lymphocytic leukemia. Therefore, inhibition of Lck by dasatinib or other small-molecule inhibitors may contribute to the treatment of glucocorticoid-resistant malignancies (Harr et al., 2010). The second-generation tyrosine kinase inhibitor (TKI) dasatinib is also used for the treatment of chronic, accelerated, or blastic phase chronic myeloid leukemia (CML) patients who are intolerant to previous treatment, including imatinib (Chen and Chen, 2015). The mechanism may be related to inhibiting Lck at low picomolar concentrations, thus suppressing T cell proliferation and activation, restraining signal transduction through the TCR complex, as well as preventing proinflammatory cytokine production (Majolini et al., 1999). In addition to controlling T cell function, proteomic analysis of nuclear proteins found that Lck was also detectable in pro-B cells (Jin et al., 2003). Ulivieri et al. (2003) indicated that Lck expression was induced when B cells were activated or stimulated by Epstein-Barr virus infection or BCR cross-linking. Increased levels of Lck have been reported in human B-1 (CD5^+^) cells, murine peritoneal B-1 cells and CLL cells (Ulivieri et al., 2003; Dal Porto et al., 2004). In B cell chronic lymphocytic leukemia (B-CLL), researches found that Lck has a vital role in potentiating BCR signaling, since siRNA knockdown of Lck expression inhibited BCR-induced CD79 phosphorylation, blocked proximal BCR signaling events and reduced overall cell survival. Expression of Lck may be related to the selective expansion of malignant B-1 cells, although not directly involved in neoplastic transformation (Majolini et al., 1998).

Activation of NF-κB in immune cells results in the production of pro-inflammatory growth factors, cytokines and chemokines such as VEGF, IL-1, IL-6 and TNF. These cytokines can further activate signal transducer and activator of transcription 3 (STAT3) in cancer cells, contributing to a tumor micro-environment that enhances survival and proliferation of cancer cells, epithelial-to-mesenchymal invasion and transition, angiogenesis and metastasis (Taniguchi and Karin, 2018). NF-κB functions as an anti-apoptotic molecule through activating the transcription of related genes, such as activating endogenous inhibitors of apoptosis [apoptosis protein1 (IAP1), IAP2, TRAF1 and TRAF2, B-cell lymphoma-2 (BCL2) family of proteins like BCL2 and BCL-X_L_] that block both death receptor-mediated apoptosis and mitochondrial pathways. The NF-κB subunit c-Rel was found to be preferentially expressed in myeloid cells and lymphocytes; c-Rel deletion in myeloid cells specifically abolished tumor growth in mice injected with lymphoma cell lines, reduced cell proliferation, and altered cell cycle progression in human melanoma cell lines (Priebe et al., 2019; Li et al., 2020). c-Rel inhibition blocked human myeloid-derived suppressor cell expansion, which was markedly increased in patients with chronic inflammation or cancer (Bronte et al., 2016; Kumar et al., 2016). NF-κB activity is upregulated in CLL (Mansouri et al., 2016), which is characterized by increased frequency of monoclonal B lymphocytes in peripheral lymphoid tissues, blood, and bone marrow caused by defective apoptosis rather than cell proliferation. NF-κB expression in CLL cells is closely connected to the tumor micro-environment (Lopez-Guerra and Colomer, 2010), which contributes to the increased survival of CLL cells. In addition, stimulation of the BCR can significantly increase constitutive activation of NF-κB in CLL cells, which in turn induces IL-6 production, leading to the successive phosphorylation of STAT3 on tyrosine residues, which is closely associated with CLL pathogenesis (Rozovski et al., 2017). CLL cells with high expression of Lck show higher levels of survival, BCR-mediated IKK, ERK and Akt phosphorylation, compared to cells with low levels of Lck expression. Lck is thought to be a vital contributor of Syk activity in CLL cells, while Syk is the critical mediator for PLCγ2 activation, which can further lead to activation of the NF-κB pathway. Lck in BCR-stimulated CLL cells can induce phosphorylation of Syk, activation of NF-κB, MAPK and PI3K/Akt signaling pathways, and enhance cell survival. Therefore, inhibition of Lck activity may be an attractive therapeutic option, and small molecule compounds that target Lck may contribute to inhibit BCR-mediated IKK phosphorylation and NF-κB activation, as well as antigen receptor-mediated activation of T cells.

#### 3.2.2 Lck-NF-κB signaling in solid cancers

In addition to the high levels of Lck expression in hematological malignancies (CLL,T-ALL, etc.), several studies have found increased expression of Lck in many solid tumors, including glioma (Summy and Gallick, 2003; Du et al., 2009; Kim et al., 2010; Zepecki et al., 2019), cholangiocarcinoma (CCA) (Pei et al., 2015; Sugihara et al., 2018; Conboy et al., 2023), and breast cancer (Bai et al., 2019; Meng et al., 2021), etc. Lck may mediate tumorigenesis and carcinogenesis in various tumors (Bommhardt et al., 2019; Han et al., 2022), suggesting that Lck may be a potential diagnostic biomarker and therapeutic target for solid cancers. Therefore, selective inhibitors targeting Lck in human tumor cells may demonstrate remarkable therapeutic effects, not only for the treatment of hematological malignancies but also in solid cancers.

Activity and expression of Src family kinases was significantly increased in normal brain tissues than in non-brain tissues (Summy and Gallick, 2003); Moreover, compared to healthy brain, the activation degree of Src family kinases is much higher in brain tumors. High expression of Src family kinases is associated with glioma cell proliferation, and their inhibition can abolish proliferation of glioma cells (Du et al., 2009). In glioblastoma, Lck expression and activated pLck^Y394^ were increased in high-grade tumors (Zepecki et al., 2019). Kim et al. (2010) reported that Lck inhibition decreased the frequency of glioma stem-like cells, while siRNA-mediated knockdown of Lck could clearly suppress expansion of CD133^+^ cells induced by fractionated radiation. Lck blocking also induced expression of glioma-initiating cell markers, as well as inhibited expression of self-renewal-related proteins (Sox2, Notch2, and b-catenin) in glioma stem cells. Furthermore, Lck inhibition restored sensitivity of glioma cells to cisplatin and etoposide, suggesting that Lck plays a critical role in glioma cell expansion induced by fractionated radiation and cellular sensitivity reduction to anticancer treatments. Another study demonstrated that the Lck inhibitor A770041 could specifically control cytoskeletal changes during glioma cell migration (Zepecki et al., 2019). Human glioblastoma treatment with small molecule inhibitor of Lck (Lck-I) can significantly restrain tumor growth, and Nanog-targeted gene expression associated with patient survival. The NFKB1 (p105/p50) subunit is a critical regulator of NF-κB activity, and innate and adaptive immune function was impaired in NFKB1 knockout mice (Artis et al., 2005). *nfkb1* ^*−/−*^ mice are characterized by enhanced inflammation and susceptibility to DNA damage, resulting in a rapid ageing phenotype, and even cancer (Cartwright et al., 2016). *NFKB1* heterozygous mutation in humans reduces the level of p105 and p50 in resting cells and depletes p105 phosphorylation, resulting in common variable immunodeficiency, which manifests as ineffective isotype class switching and inferior antibody responses in patients (Boztug et al., 2016). In addition, low expression of Kip1 ubiquitination-promoting complex 1 (KPC1), a ubiquitin ligase required for p105 processing to generate p50, was correlated to p50 reduction and glioblastoma incidence in humans (Cartwright et al., 2016).

Studies demonstrated that the expression of Lck is not only evident in enormal human cholangiocytes, but also in the cholangiocarcinoma (CCA) cell lines and the patient-derived xenograft models (Sugihara et al., 2018). In CCA, Lck may be identified as a drug target, because targeting Lck can decrease viability of CCA cells both *in vivo* and *in vitro*. The Hippo signaling pathway regulates cell proliferation and apoptosis, organ size, and tissue homeostasis through inhibiting activation of Yes-associated protein (YAP). Overexpression of YAP can be detected in most CCA cases, and YAP is localized in the nucleus where it can drive oncogenic gene expression programs (Pei et al., 2015). Lck can phosphorylate YAP on tyrosine 357; YAP transcriptional activity and tyrosine phosphorylation are both decreased with siRNA targeted knockdown of Lck. It has been reported that dasatinib could suppress tyrosine phosphorylation of YAP and *in vivo* growth of CCA patient-derived xenografts (Sugihara et al., 2018). NTRC 0652-0, a novel selective small molecule inhibitor of Lck, can reduce proliferation and enhance apoptosis of CCA tumor cells, possibly through suppression of YAP^Y357^ phosphorylation, increased YAP cytoplasmic localization, and increased cleaved caspase 3 levels in tumors (Conboy et al., 2023).

Compared to healthy control samples, Lck was significantly overexpressed in breast cancer tissues and its overexpression was related to worse survival outcomes and poor prognosis (Bai et al., 2019; Meng et al., 2021). Meng et al. (2021) reported that infiltration of CD4^+^ T cells, CD8^+^ T cells, B cells, and dendritic cells was positively correlated with Lck activity, but Lck was negatively correlated with tumor purity. The Lck inhibitor dasatinib could reduce bone metastases of breast cancer, while it could also be clinically therapeutic for breast cancer patients that overexpress HER2 (Montero et al., 2011).

Lck acts both as an adaptor molecule and tyrosine kinase, which can bind to and phosphorylate a great number of cell surface and intracellular substrates during T cell activation. B cells require Lck to enter the S phase since many germinal center B cells are proliferating (Taieb et al., 1993). However, lower or higher expression of Lck may have unwanted consequences. Expression of Lck is high in RA, CLL, and glioma, suggesting that it may contribute to the pathogenesis of certain diseases. Lck-deficient lymphoid cells are resistant to apoptosis induced by chemotherapeutic agents, a phenotype that is reversed when Lck is re-expressed (Samraj et al., 2006). In short, regulating the abnormal expression and activation of Lck should be considered in the treatment of autoimmune diseases and relevant malignancies.

Dasatinib (Sprycel) is an ATP competitor that has been employed in the therapy of Philadelphia chromosome-positive chronic myelogenous leukemia and imatinib-resistant chronic myelogenous leukemia (Lee et al., 2010; Wang et al., 2013). Dasatinib suppresses the function and activation of T cells at clinically relevant concentrations mainly through Lck inhibition, and it can bind to the ATP-binding site of Lck and induce an inactive conformation structure, thus reducing the level of Tyr^394^ phosphorylation. Upon dasatinib treatment, phosphorylation of the Lck substrate Zap-70 decreased, and secretion of cytokines including IL-2, TNF-α, and IFN-γ was also rapidly abolished (Lee et al., 2010; Leclercq et al., 2021). Dasatinib is also indicated for T-ALL treatment through inhibiting Lck function by interrupting downstream propagation of TCR signals, thus leading to suppression of cell proliferation (Laukkanen et al., 2022). In addition, Han et al. (2014) found that dasatinib could significantly inhibit migration of glioma stem cells. Adoptive immunotherapy with genetically engineered chimeric antigen receptor (CAR) T cells is a revolutionary method for cancer treatment, although this single-shot “living drug” treatment is essentially out of control after administration (Sterner and Sterner, 2021). Dasatinib can block the ATP binding sites of Lck (Blake et al., 2008), thus intercepting CAR signaling and immediately blocking CD4^+^ and CD8^+^ CAR T cell function, while it can also control cytokine secretion and cytolytic activity. Moreover, dasatinib does not affect CAR T cell viability, and this block in T cell function is completely reversible after dasatinib discontinuation (Mestermann et al., 2019). Therefore, dasatinib may be regarded as a widely applicable pharmacologic on/off switch in CAR T cells.

Small molecule bioactives that possess inherent fluorescent properties are considered as incredibly valuable tools for exploring fundamental mechanisms in healthy and diseased cells and tissues (Klymchenko, 2017). Prodan is a fluorophore that has been widely used as a probe in several biological systems because of its desirable spectral performance. Fluorescent properties can be achieved by including Prodan-derived fluorophores into pharmacophores of the ATP-competitive kinase inhibitor. Fluorescent Lck inhibitor exhibits good solvatochromic capability after binding to the kinase, and the fluorescence intensity of the inhibitor is correlated with the concentration of Lck. The Prodan-derived Lck inhibitor could be regarded as a ponderable molecular tool for real-time studies of intracellular Lck signaling, because of its particular solvatochromic properties, which allowed users to distinguish between bound and unbound molecules in the cellular environment (Fleming et al., 2019).

## 4 Conclusion and perspective

It is widely known that activation of NF-κB is commonly involved in numerous human diseases, and contributes to further deterioration. Numerous stimuli can cause activation of NF-κB. However, global therapeutic inhibition of NF-κB may lead to unexpected outcomes, which are presumably thought to be one of the main reasons why targeting NF-κB pathway has failed in clinical trials (Afonina et al., 2017). Further insight into the biochemical, pathophysiological, and functional interactions between NF-κB and other signaling pathways may be useful to avoid such failures. Lck and NF-κB are both valuable for cell development and differentiation. In this review, we introduced the Lck-NF-κB signaling pathway, exploring its importance for the pathogenesis of different disorders. In autoimmune diseases, the specific Src kinase inhibitor damnacanthal could significantly inhibit serine phosphorylation and subsequent degradation induced by IκB-α. Inhibition of Lck and Zap-70 could prevent both NF-κB activation and tyrosine phosphorylation of IκB-α. In BCR-stimulated CLL cells, Lck has the ability to induce phosphorylation of Syk, activation of NF-κB, MAPK and PI3K/Akt signaling pathways and enhance cell survival. Biochanin also prevented cell activation, which contributed to autoimmune disorders and malignancy, since it blocked NF-κB activation through preventing not only upstream IKK, but also PTK activity (Manna, 2012). Src kinases, especially Lck, may be considered as useful targets for blocking over-activation of NF-κB, thus treating related diseases, such as autoimmune disorders and malignancies. Patients with inflammatory diseases or those subjected to organ transplantation would benefit from drugs that selectively inhibit Lck. However, since correlational studies about the Lck-NF-κB signaling pathway are limited, there is still insufficient evidence to determine which proteins or molecules in this pathway may be essential for disease treatment. Therefore, our review aimed to provide a basis to determine appropriate targets in the Lck-NF-κB signaling pathway to effectively design therapeutic strategies against autoimmune diseases and malignancies.

## Glossary

### The following abbreviations are used in this document:

A-loop: the activation loop
BAFFR: B cell activation factor receptor
BCR: B cell antigen receptor
B-CLL: B cell chronic lymphocytic leukemia
CAR: chimeric antigen receptor
CLL: human chronic lymphocytic leukemia
CIA: collagen-induced arthritis
CML: chronic myeloid leukemia
Csk: C-terminal Src kinase
DCs: dendritic cells
FcR: Fcε receptor
FLS: fibroblast-like synoviocytes
GPI: glycosylphosphatidylinositol
IAP1: inhibitor of apoptosis protein1
IFN-γ: interferon-γ
IKK: IκB kinase
IL: intercleukin
IP3: inositol 1,4,5-Trisphosphate
ITAM: immunoreceptor tyrosine-based activation motifs
JAK: Janus kinase
KPC1: Kip1 ubiquitination-promoting complex 1
LAT: family adaptor molecule linker for activation of T cells
Lck: lymphocyte-specific protein tyrosine kinase
MAPK: Mitogen-Activated Protein Kinase
MDSCs: myeloid-derived suppressor cells
MMP-9: matrix metallopeptidase 9
MSK1: mitogen- and stress-activated kinase 1
NF-κB: Nuclear factor-κB
NIK: NF-κB-inducing kinase
NBS: Nijmegen breakage syndrome
OP: osteoporosis
PDK1: 3-phosphoinositide-dependent kinase 1
PH: pleckstrin homology
PI3K: phosphatidylinositol-3-kinase
PIP2: phosphatidylinositol 4,5-biphosphate
PIP3: phosphatidylinositol 3,4,5-triphosphate
PKC: protein kinase Cβ
PKR: double-stranded RNA-activated protein kinase
PLCγ1: phospholipase C-γ1
PMNs: polymorphonuclear neutrophils
PTEN: phosphatase and tensin homology
PTK: protein tyrosine kinase
RA: rheumatoid arthritis
SFK: Src family kinase
SLE: systemic lupus erythematosus
SLP-76: SH2 domain containing leukocyte protein of 76 kDa
STAT3: signal transducer and activator of transcription 3
Syk: spleen tyrosine kinase
TAK1: TGF-β-activated kinase-1
T-ALL: acute T cell lymphocytic leukemia
TCR: T cell antigen receptor
Th: helper T cells
TKI: tyrosine kinase inhibitor
TLR ligands: toll-like receptor family
TNF-α: tumor necrosis factor-α
TRAF6: TNF receptor-associated factor-6
Treg: regulatory T cells
uPA: urokinase type plasminogen activator
VEGF: vascular endothelial growth factor
YAP: Yes-associated protein;
ZAP70: Zeta-chain-associated protein kinase 70.
